# Novel strategies to target chemoresistant triple-negative breast cancer

**DOI:** 10.18632/genesandcancer.204

**Published:** 2020-07-22

**Authors:** Jaganathan Venkatesh, Arun K. Rishi, Kaladhar B. Reddy

**Affiliations:** ^1^ John D. Dingell VA Medical Center, Wayne State University, Detroit, MI, USA; ^2^ Department of Oncology, Wayne State University, Detroit, MI, USA; ^3^ Karmanos Cancer Institute, Wayne State University, Detroit, MI, USA; ^4^ Department of Pathology, Wayne State University, Detroit, MI, USA

**Keywords:** Cell cycle and apoptosis regulator 1 (CARP-1), cancer stem cells, CFM-4.16, apoptosis, triple-negative breast cancer

## Abstract

Previous studies from our group and others have shown that current drug treatment(s) strategies eliminate bulk of tumor cells (non-CSCs) but it had a minimal effect on cancer stem cells (CSCs) leading to resistance and tumor recurrence. We studied the effects of CFM-4.16 (CARP-1 functional mimetic) and/or cisplatin on four Triple-negative breast cancer (TNBC) MDA-MB-468, MDA-MB-231, CRL-2335 and BR-1126, two cisplatin resistant CisR/MDA-231 and CisR/MDA-468 and cancer stem cells (CSCs) from resistant cell lines. TNBC cells treated with CFM-4.16 plus cisplatin inhibited the expression of FZD8, LRP6 and c-Myc and significantly enhanced cell death in all the cell lines by ~70%-80% compared with the control(s). When Cisplatin resistant CisR/MDA-231 and CisR/MDA-468 were treated with CFM-4.16 plus cisplatin, they also showed a reduction in FZD8 and LRP6 and increased apoptosis compared to control group. Similarly, CFM-4.16 plus cisplatin treatment reduced mammospheres formation abilities of CSCs by 80-90% compared to control group, increased PARP cleavage and apoptosis. Data shows CFM-4.16 plus cisplatin treatment significantly increased apoptosis/cell death in parental, cisplatin resistant and CSCs. Taken together the data suggests that FZD8-mediated Wnt-signaling plays a major role in mediating CSCs growth and resistance to chemotherapy and its inhibition enhances the chemotherapeutic response in TNBC.

## INTRODUCTION

Triple-negative breast cancer (TNBC) continues to be a major health problem worldwide despite recent advances in its diagnosis and treatment. Chemotherapy is currently the only systemic treatment option for TNBC, but optimal treatment protocols are yet to be established [1, 2]. Taxane and anthracycline-based regimens represent the mainstay in TNBC therapy, while platinum-based chemotherapy has shown promising results in the neoadjuvant and metastatic settings [1]. Despite the aggressive nature of TNBC, 20% of patients present a pathologic complete response (pCR) after neoadjuvant chemotherapy [3]. The differences in clinical outcomes following neoadjuvant treatment imply that a subset of TNBCs is sensitive to chemotherapy while others become resistant during treatment or are intrinsically less susceptible. In breast cancer, both *in vivo* and patient data showed a substantial increase in cancer stem cells (CSC) in the residual tumors following treatment with conventional chemotherapy [4, 5]. Many studies support the notion of CSC importance in TNBC, such as the positive correlation observed between the expression of CSC markers (CD44, ALDH1) and lower survival rates of TNBC patients [6, 7]. Accumulating data also suggested the chemoresistant CSCs may be a major factor in TNBC relapse [8, 9]. We postulate inhibition of factors crucial for CSC maintenance such as Wnt-signaling can sensitize TNBC cells to chemotherapy.

There is compelling evidence indicating that aberrant activation of Wnt/β-catenin signaling leads to mammary carcinogenesis [10 -12]. Enhanced nuclear/cytoplasmic β-catenin staining was found in ~ 60% of human breast cancer tissues [12, 13] suggesting that Wnt-signaling is activated in these tumors. Overexpression of Wnts and FZDs or constitutively active FZD regulates various processes, which play a major role in tumor growth, CSCs and metastasis [14-16]. Frizzled-8 receptor (FZD8) expression was significantly higher in human breast cancer tissues compared with the adjacent normal tissues, and higher expression of FZD8 was closely correlated with lymph node metastasis [17]. We have previously shown that residual tumors that survived chemotherapy show increased FZD8 and CSCs in TNBC tumors and cell lines compared to control group [5]. Clinically Low-density lipoprotein receptor-related protein 6 receptor (LRP6) is an essential Wnt co-receptor of the Wnt/β-catenin signaling pathway. LRP6 was shown to be significantly elevated in ~25% of breast cancers more so in TNBC than other types of breast cancer [18]. However, mutations in Wnt pathway components such as Adenomatous polyposis (APC), ctnnb1(encoding β-catenin), Axin, etc., are rarely detected in human breast cancer [10, 13, 19]. LRP6, but not LRP5, is frequently up-regulated in human breast carcinomas [18]. Together, these data suggested that Wnt/β-catenin signaling mediated through FZD8 and LRP6 plays a major role in human breast cancers, drug resistance and metastasis [2, 14, 18, 20].

Cell cycle and apoptosis regulator 1 (CARP-1/CCAR1) is a peri-nuclear phospho-protein, that regulates cell growth and apoptosis signaling in a variety of cancer cells [21, 22]. CARP-1 expression is often elevated in cells experiencing stress due to growth factor withdrawal or chemotherapy-induced cell cycle arrest and apoptosis [21, 22]. Knockdown of CARP-1 resulted in resistance to apoptosis by ADR or EGFR tyrosine kinase inhibitors demonstrating the requirement of CARP-1 in cell growth inhibition and apoptosis signaling by these agents [21-23]. Breast tumors have been shown to have much lower CARP-1 compared to surrounding tissues. A chemical biology based high-throughput screening of a chemical library resulted in identification of a number of novel, small molecular inhibitors of CARP-1 binding with APC/C subunit of APC2 [24]. It was previously shown that CFM-4 is a CARP-1 functional mimetics (CFMs), and blocks its interaction with APC2, cause G2M cell cycle arrest, and inhibits cell growth by inducing apoptosis in various cancers. In contrast, CFM-4 treatment of an immortalized non-cancerous cell line MCF10A had a minimal effect on growth [24, 25]. Previous studies have also found that CFM-4 analog and CFM-4.16 binds with CARP-1, and causes elevated levels of CARP-1, stimulates apoptosis [24, 26, 27].

## RESULTS

### CFM-4.16 plus cisplatin enhances cell death in TNBC cell lines

**Figure 1:**
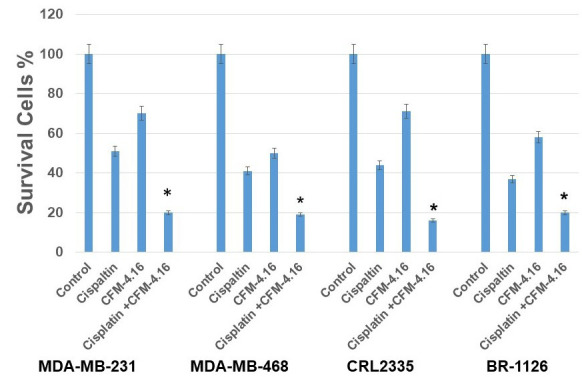
CFM-4.16 plus cisplatin treatment resulted in a significantly enhanced cell death in multiple TNBC cell lines: MDA-MB-231, MDA-MB-468, CRL2335 and BR-1126 (PDX derived TNBC cell line) were treated with CFM-4.16 (10 µM), cisplatin (10µg/ml) or CFM-4.16 (10 µM) plus cisplatin (10µg/ml) for 24 hours. Cell viability was then analyzed by MTT method. CFM-4.16 plus cisplatin treatment significantly inhibited MDA-MB-231, MDA-MB-468, CRL2335 and BR-1126 compared to their control group (*p <0.05). Each point represents the mean + S.E. of triplicate determination.

**Figure 2:**
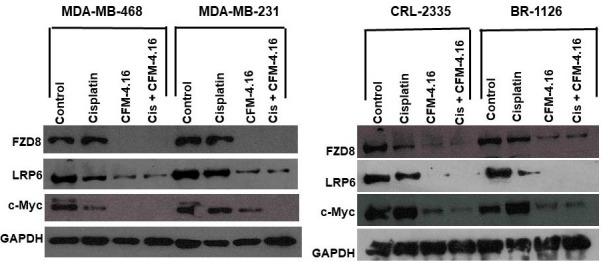
Expression of FZD8, LRP6 and c-Myc in MDA-MB-231, MDA-MB-468, CRL2335 and BR-1126 TNBC cells treated with CFM-4.16, cisplatin or CFM-4.16 plus cisplatin for 24 hours. Whole-cell extracts were prepared and analyzed for FZD8, LRP6 and c-Myc by Western blotting. Equal protein loading was compared with that of GAPDH

We used four cell lines; three TNBC cell lines MDA-MB-468, MDA-MB-231, CRL-2335 and one BR-1126 (PDX derived TNBC cell line) to determine the effect of CFM-4.16, cisplatin, or combination on cell death. The MTT data indicate that CFM-4.16 or cisplatin induced approximately 30%-50% cell death in TNBC cell lines. However, combination of CFM-4.16 plus cisplatin enhanced cell death to approximately ~80% in TNBC cell lines (Figure 1). Our group and others have previously shown that Wnt-signaling plays a major role in tumor growth and drug resistance in TNBC [5, 28-31]. Western blot analysis showed that cisplatin had varying levels of inhibition(s) on FZD8, LRP6 and c-Myc. In contrast, CFM-4.16 treatment shows consistent protein reduction in FZD8, LRP6 and c-Myc. However, combination of CFM-4.16 plus cisplatin treatment resulted in consistent reduction in all of the above proteins (Figure2). Taken together, these data suggest that CFM-4.16 plus cisplatin treatment had a significant effect in reducing levels of major Wnt-signaling proteins and this leads to enhanced cell death in all the four TNBC cell lines.

### Effect of CFM-4.16 plus cisplatin on cisplatin-resistant TNBC cell lines

**Figure 3:**
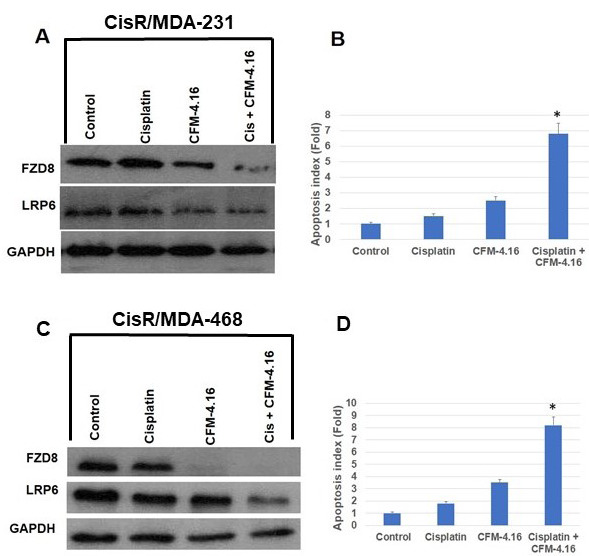
Effect of CFM-4.16 plus cisplatin on CisR/MDA-231 and CisR/MDA-468 TNBC cells. CisR/MDA-231 and CisR/MDA-468 TNBC cells were treated with CFM-4.16 (10 µM), cisplatin (10µg/ml) or CFM-4.16 (10 µM) plus cisplatin (10µg/ml) for 24 hours. Whole-cell extracts were prepared and analyzed for FZD8 and LRP6 by Western blotting A., C. Equal protein loading was compared with that of GAPDH. Under similar conditions, apoptosis was quantified by Cell Death ELISA and normalized to values measured in untreated cells. CisR/MDA-231 and CisR/MDA-468 TNBC cells showed a significant increase in apoptosis in the presence of CFM-4.16 plus cisplatin in comparison to untreated cells B., D. (*p <0.001). Data are the mean + SE of triplicate determinations.

Cisplatin is one of the most successful anticancer agents used in TNBC and other tumors [32, 33]. Overcoming resistance to chemotherapeutic agents would represent a major advance in clinical management of breast cancer. We tested the efficacy of CFM-4.16 plus cisplatin on cisplatin resistant TNBC cells. We developed a cisplatin resistance phenotype in MDA-231 and MDA-468 TNBC cells, and this was achieved through growth of the cells over time in the presence of increasing concentrations of cisplatin. To determine if CFM-4.16 plus cisplatin enhances cell death in cisplatin-resistant MDA-MB-468 (CisR/MDA-468) and cisplatin-resistant MDA-MB-231 (CisR/MDA-231) cells, we treated cells with cisplatin, CFM-4.16 or combination for 24-hours. The data in Figure 3A and 3C indicates that cisplatin had no or minimal effect on FZD8 or LRP6. However, CFM-4.16 significantly inhibited FZD8 in CisR/MDA-468 cells but has a moderate effect on CisR/MDA-231 cells. However, combination of CFM-4.16 plus cisplatin had a significant effect on FZD8 and LRP6 reduction compared to control group, and significantly enhanced apoptosis (Figure3B&3D). These results suggest that CFM-4.16 plus cisplatin treatment overcomes cisplatin resistance in TNBC cells.

### CFM-4.16 plus cisplatin on CSCs derived from cisplatin-resistant TNBC cells

Emerging evidence suggests that CSCs, which have tumor-initiating potential and self-renewal capacity, may be responsible for poor outcome by promoting therapy resistance, recurrence and metastasis [5, 9, 34, 35]. It was previously shown that the cell fraction with the CD44+/CD24-/Lin- phenotype in breast cancer patient tissues could recapitulate tumor burden in mice [36]. Later, it was also shown that a subpopulation of cells with high aldehyde dehydrogenase (ALDH) activity could initiate tumors *in vivo* [37]. Since then, the CD44+/CD24- phenotype and high ALDH activity have become the “gold standard” signature for breast cancer stem cells. Different group, including our group have shown that chemotherapy can eliminate the bulk of tumor cells (non-CSCs), but it failed to eliminate CSCs, thereby making these cells the leading cause of therapy resistance and recurrence [5, 34, 38, 39].

**Figure 4:**
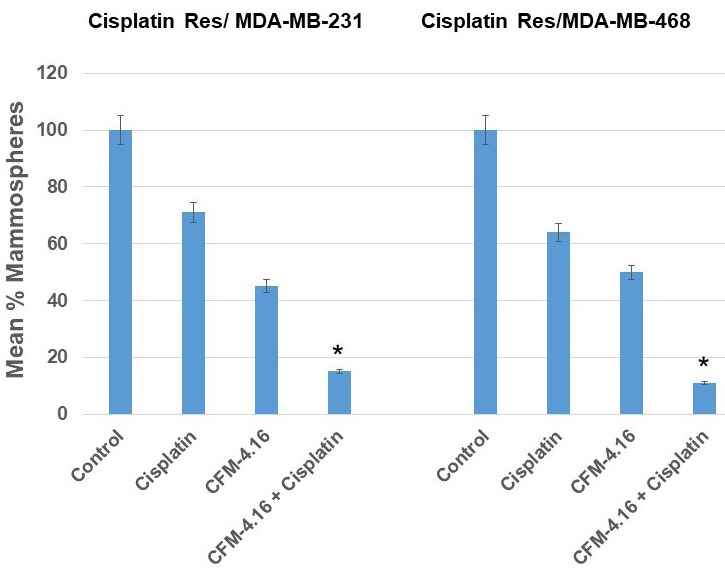
Effect of CFM-4.16 plus cisplatin treatment on the ability of cancer stem cells to form mammospheres. The *in vitro* mammospheres was quantified by counting the mammospheres after treatment with CFM-4.16, cisplatin or CFM-4.16 plus cisplatin compared to controls. CFM-4.16 plus cisplatin treatment had maximum inhibition on cancer stem cell’s ability to form mammospheres in CisR/MDA-231 and CisR/MDA-468 derived CSCs compared to their control group (*p < 0.05). Data are mean + SE of triplicate determinations.

One of the major characteristic features of CSCs is their ability to form mammospheres in suspension culture [39, 40]. We have previously shown that after two passages of mammospheres in culture, cells derived from mammospheres showed ~90% positivity for ALDH by flow cytometer, and these cells have the ability to form tumors in mice (data not shown). In this study, we used cells derived from mammospheres after two passages as CSCs. We investigated the effect of cisplatin, CFM-4.16 or its combination on mammosphere formation using CisR/MDA-231 and CisR/MDA-468 TNBC cell lines. The results presented in Figure 4 shows that cisplatin treatment reduced mammosphere formation ability of CSCs by ~ 30% compared to an untreated control group in CisR/MDA-231 cells, CFM-4.16 treatment reduced mammosphere formation by ~ 55%, and the combination of CFM-4.16 plus cisplatin reduced mammosphere formation ability by ~85% compared to control group. Similarly, in CisR/MDA-468 derived CSCs, cisplatin treatment reduced mammosphere formation ability by ~36%, CFM-4.16 by ~59% and combination reduced mammosphere formation ability by 89% (Figure 4).

**Figure 5:**
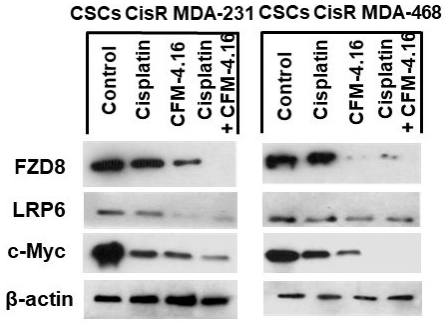
Effect of CFM-4.16 plus cisplatin treatment on the expression of FZD8, LRP6 and c-Myc in CisR/MDA-231 and CisR/MDA-468 derived cancer stem cells. Cell extracts were prepared and analyzed for FZD8, LRP6 and c-Myc by Western blotting. Equal protein loading was compared with that of β-actin.

**Figure 6:**
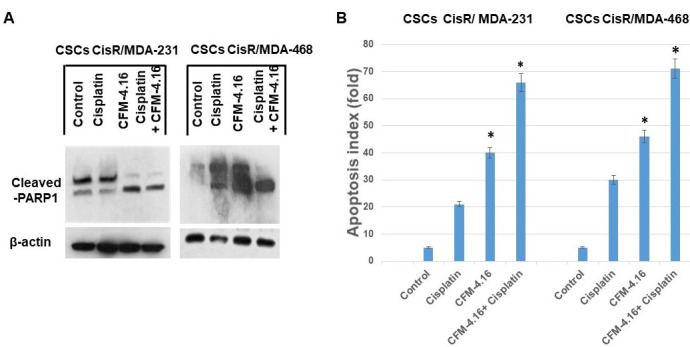
CFM-4.16 plus cisplatin increases PARP-cleavage and apoptosis in CisR/MDA-231 and CisR/MDA-468 derived cancer stem cells. CisR/MDA-231 and CisR/MDA-468 derived cancer stem cells were treated with CFM-4.16, cisplatin or CFM-4.16 plus cisplatin for 24 hours. A. Cancer stem cell extracts were prepared and analyzed for PAPR cleavage by Western blotting. Equal protein loading was compared with that of β-actin. B. Under similar conditions, apoptosis was quantified by Cell Death ELISA and normalized to values measured in untreated cells. Cancer stem cells derived from CisR/MDA-231 and CisR/MDA-468 TNBC cells showed a significant increase in apoptosis in the presence of CFM-4.16 plus cisplatin in comparison to untreated cells (*p < 0.001). Data are the mean + SE of triplicate determinations.

The effect of CFM-4.16, cisplatin or CFM-4.16 plus cisplatin treatment was also examined on Wnt-signaling proteins and apoptosis in CSCs derived from cisplatin resistant TNBC cells. The results in Figure 5 show that cisplatin had minimal inhibition on FZD8, LRP6 protein levels in CisR/MDA-231 and CisR/MDA-468 derived CSCs. CFM-4.16 treatment, on the other hand, causes a reduction in FZD-8, LRP6 proteins in both CisR/MDA-231 and CisR/MDA-468 derived CSCs. However, combination of CFM-4.16 plus cisplatin treatment resulted in consistent reduction in all the proteins (Figure5). Western blot(s) showed a significant increase in PARP cleavage (Figure6A) and cell death in CSCs (Figure6B) derived from cisplatin-resistant TNBC cell lines. Taken together, these data suggest that CFM-4.16 plus cisplatin treatment can reduce FZD8, and LRP in parental, cisplatin resistant and CSCs derived from cis-resistant TNBC cells and also increase apoptosis/cell death. These findings suggest that CFM-4.16 plus cisplatin therapy has the potential to improve therapeutic outcome in TNBC patients.

## DISCUSSION

Abnormal activation of Wnt-signaling has been implicated in the regulation of CSCs including breast cancer, lung cancer, colorectal cancer, etc. [11, 12, 41, 42]. CSCs were shown to be responsible for tumor initiation, growth, chemotherapeutic resistance and recurrence [9, 34, 43, 44]. The propensity of CSCs to resist chemotherapy, thereby maintaining a pool of tumorigenic cells from which tumor(s) can reoccur [5, 45, 46]. We hypothesized that treatment strategies that target both CSCs and non-CSC (bulk of the tumor) could improve therapeutic outcome. The signaling pathways that regulate self-renewal and differentiation of CSCs are not well understood. A number of studies support functional versatility of Wnt/β-catenin signaling in CSCs and non-CSC’s [2, 28, 29, 43]. The results from the present investigations clearly show that MDA-MB-231, MDA-MB-468, CRL-2335 and BR-1126 TNBC cells treated with CFM-4.16 plus cisplatin show significantly enhanced cell death (~ 70-80%) as compared to untreated cells (Figure 1). The results from this study have also shown that treatment with CFM-4.16 alone caused significant reduction in FZD8, LRP6 and c-myc (Figure 2) and induced ~30-40% cell death. Cisplatin alone had a marginal effect on FZD8 and LRP6 and induced ~ 40-50% cell death. However, combination of cisplatin plus CFM-4.16 increased the cell death to ~70-80% in all the four TNBC cell lines. Taken together, the data suggest the possibility that restoration of CARP-1 through functional mimetic CFM-4.16 reduces Wnt-signaling and significantly increases apoptosis in the presence of cisplatin.

Chemotherapy resistance presents a significant hurdle for successful treatment in TNBC. Platinum-based chemotherapy has shown promising results in TNBC, they frequently develop chemoresistance [2 ,9]. To determine if CFM-4.16 plus cisplatin enhances cell death in cisplatin-resistant MDA-MB-468 (CisR/MDA-468) and cisplatin-resistant MDA-MB-231 (CisR/MDA-231) cells were treated with cisplatin, CFM-4.16 or combination. Our data shows a combination of CFM-4.16 plus cisplatin significantly reduced Wnt-signaling through inhibition of FZD8 and LRP6 and enhanced apoptosis (Figure 3B&3D). These results suggest that CFM-4.16 plus cisplatin treatment overcomes cisplatin resistance in TNBC cells.

Successive studies detected a higher percentage of CSCs in primary breast tumors following neoadjuvant chemotherapy [4, 5, 8]. Different groups, including our group showed that CSCs are resistant to chemotherapy, leading to enrichement of CSCs in residual tumor both *in vitro* and *in vivo* [4, 5]. Wnt-signaling was implicated in CSC’s self-renewal. Therefore, targeting Wnt-signaling in CSCs may be a promising therapeutic approach for TNBC. To test this premise we treated the CSCs derived from cisplatin-resistant cells with CFM-4.16, cisplatin and CFM-4.16 plus cisplatin and determined the mammosphere formation. This assay has been used as a surrogate reporter of CSCs activity in mammary gland [36, 40, 47]. The results from this study showed a combination of CFM-4.16 plus cisplatin reduced mammosphere formation ability by ~85-90% when compared to control group (Figure 4). Our data also showed that proteins extracted from the treated mammosphere had significant reduction in Wnt-signaling proteins FZD8, LRP6, c-Myc and increased PARP cleavage leading to apoptosis in CSCs compared to control group.

Previous studies from our group and others have shown that activation of Wnt-signaling leads to increased expression of c-Myc through β-catenin [19–21]. We investigated the role of c-Myc in regulation of Wnt signaling and its effect on CSCs and response to chemotherapy. We overexpressed c-Myc in MDA-MB-468 (Myc/MDA-468) and MDA-MB-231 (Myc/MDA-231) TNBC cells. Myc overexpression resulted in a significant increase in FZD8 compared to vector transfected control cells [31]. Based on these observations and others, we propose that a positive feedback loop exists between Myc, and FZD8 breast cancer cells. Myc also controls the expression of numerous genes, which are essential to regulate EMT and/or CSCs. Our data showed over expression of c-Myc in the Myc/MDA-468 cells increased expression of ALDH, a marker for CSCs, while it had a minimal effect on EMT regulating proteins such as Vimentin and Zeb 1. In contrast, Myc/MDA-231 cells showed increased levels of Vimentin, and Zeb 1 a marker for EMT changes when compared to vector-expressing controls, and ALDH levels were undetectable by Western blot analysis [31]. In addition, we have also demonstrated that Myc/MDA-468 cells, exhibit higher CSCs levels and are resistant to cisplatin plus Iniparib (PARP inhibitor), paclitaxel, docetaxel and iniparib alone. However, Myc/MDA-231 cells that showed increased EMT changes were sensitive to combination therapy of cisplatin plus Iniparib. Interestingly, Myc/MDA-231 cells were resistant to chemotherapeutic agents paclitaxel, docetaxel and iniparib that are commonly used for clinical practice to treat breast cancer [31]. In contrast, combination of CFM.4.16 and cisplatin significantly increased apoptosis in both c-Myc/MDA-468 and c-Myc/MDA-231 compared to respective vector expressing control cells. This data suggests CFM-4.16 caused reduction in FZD8, LRP6 and c-Myc sensitizes cells to cisplatin.

Many studies support the notion of CSCs significance in TNBC, such as the positive correlation observed between the expression of CSC markers (CD44, ALDH1) and lower survival rates of TNBC patients [6, 8]. Eradication and/or reduction of CSCs may represent an effective anticancer therapeutic strategy. Extensive literature highlights the key role of deregulated Wnt/β-catenin signaling in TNBC and it’s more likely to develop chemotherapeutic resistance and distant metastases [5, 48, 49]. Recently numerous Wnt signaling inhibitors, including biological and small molecular agents such as PRI-724 (Dishevelled inhibitor), Ipafricept (Fc-Frizzled 8 receptor), Vantictuman (Anti-Frizzled 7 receptor), LGK974 (Porcupine inhibitor), CGP049090 (β-catenin-TCF), etc [44, 50]. However, due to the inherent heterogeneity of CSCs the targeting a single molecule may not be an effective strategy. Markers expressed on CSCs may also be displayed by normal stem cells, which limits accuracy of targeted treatment in TNBC. Therefore, we targeted Wnt-signaling by combination therapy, which was efficient in elimination both CSCs and non-CSC’s (bulk of tumor cells). Considering the complexity and diversity of TNBC, eradicating all cancer cells using one strategy is challenging task. Thus, using a combination treatment with CFM-4.16 plus cisplatin induce the synergistic effects that induced apoptosis in non-CSCs, cisplatin resistant-CSCs and -tumor cells. Recent studies suggest that CSCs are enriched after chemotherapy because CSCs are resistant to conventional chemotherapy leading to recurrence and eventual metastasis. In conclusion, our data show CFM-4.16 plus cisplatin treatment increases apoptosis/cell death in parental TNBC, cisplatin resistant and CSCs cells derived from cisplatin resistant TNBC cells. Overcoming resistance to chemotherapeutic agents would represent a major advance in clinical management of breast cancer. Taken together the data suggest that FZD8-mediated Wnt-signaling plays a major role in mediating CSCs growth and resistance to chemotherapy and inhibition of this pathway enhances the chemotherapeutic response in TNBC.

## MATERIALS AND METHODS

### Cell lines and reagents

The human triple-negative breast cell lines CRL2335, MDA-MB-231. MDA-MB-468 cells were obtained from the American Type Culture Collection (ATCC). BR-1126 (PDX derived TNBC cells) obtained from Dr. Rishi (VA and Karmanos Cancer Institute, Wayne State University). Cells were maintained in DMEM culture media. All cells obtained from ATCC were immediately expanded and frozen down such that all cell lines could be restarted every 3-4 months from a frozen vial of the same batch of cells, and authentication was done. Cisplatin was purchased from Sigma (St. Louis, MO). CFM-4.16 was obtained from Dr. Rishi (Karmanos Cancer Institute). Reagents for protein analysis and protein gel electrophoresis were obtained from Bio-Rad (Hercules, CA). All other chemicals, unless otherwise specified, were purchased from Sigma in the highest suitable purities.

### Generation of drug-resistant TNBC cells

Human TNBC MDA-MD-231 and MDA-MB-468 were cultured in the chronic presence of cisplatin. They were initially cultured in the presence of 1 µg/ml cisplatin for 2-3 weeks, and the dose was escalated to 4 µg/ml over a period of 3-4 weeks for each dose until resistance developed, and cells became adapted to routine culture in the presence of cisplatin.

### Mammosphere culture

Mammosphere culture was performed as described by Dontu *et al.* (19) with minor modification. In brief, cis/MDA-231 or cis/MDA-468 mammospheres were cultured in suspen­sion (1,000 cells/ml) in serum-free DMEM media, supplemented with B27 (1:100; Invitrogen), N2 (1:50; Invitrogen) and 10 ng/ml of EGF. For mammosphere formation assay, cultured mammospheres were enzymatically dissociated by incubation in a trypsin-EDTA solution (Invitrogen) at 37˚C. Cells were plated at 4000 cells per well of six-well ultra low-attachment plate (Corning, MA). Mammospheres were counted after 5-7 days. Mammosphere counting: Mammospheres were centrifuged and transferred to a 96-well flat bottomed plate in 100 μl of the media and counted using a microscope under low magnification. Experiments were done in triplicate.

### MTT assay

In brief, 5x104 cells were added in 96-well plate. After 24 h, cells were treated with CFM-4.16 (10 µM) plus cisplatin (10 μg/ml), CFM-4.16 (10 µM) plus cisplatin (10 μg/ml) another 24 h. Following treatments, 100 μl of 3-(4,5-dimethylthiazol-2-yl)-2,5-diphenyltetrazolium bromide (MTT) (1 mg/ml) was added into each sample and incubated for 3 h under 5% CO2 and 37˚C. The cell viability was measured by MTT, which is converted by succinate dehydrogenase in mitochondria of viable cells to yield a purple formazan dye. The formazan dye was dissolved in dimethyl sulfoxide (DMSO) and measured by absorption at a wavelength of 550 nm using Benchmark® microplate reader from Bio-Rad.

### Western blot analysis

TNBC or CSCs derived from TNBC cells were treated with CFM-4.16 (10 µM), cisplatin (10 μg/ml), or CFM-4.16 plus cisplatin for 24 h. Cells were lysed and Western blotting was performed as described previously [31] using a standard protocol. In brief, cell extracts were obtained by lysing the cells in RIPA buffer (20 mM Hepes, 100 mM NaCl, 0.1% SDS, 1% Nonidet P-40, 1% deoxycholate, 1 mM Na3VO4, 1 mM EGTA, 50 mM NaF, 10% glycerol, 1 mM EDTA, 1 mM phenylmethylsulfonyl fluoride, and 1x protease inhibitor mixture) (all reagents from Sigma). Samples containing 30-50 µg of total protein was electrophoresed on 8 % SDS-polyacrylamide gels and transferred to PVDF membrane by electroblotting. Membranes were probed with specific antibodies against FZD8 (Aviva System Biology), LRP6, c-Myc, GAPDH (Cell Signaling Technology), β-actin (ThermoFisher) followed by HRP-conjugated mouse or rabbit secondary antibodies (Amersham) accordingly. The specific bands were developed on autoradiography films by treatment of the membranes with an enhanced chemiluminescent substrate (Pierce).

### Apoptosis assay

Apoptosis was assessed using the Cell Death Detection ELISAplus kit (Sigma-aldrich) according to the manufacturer’s instructions. In brief, mammospheres were dissociated and 2X104 cells were plated in 96 well ultra-low attachment plates. Cells were treated with CFM-4.16, cisplatin or CFM-4.16 plus cisplatin for 24 h. Apoptosis was quantified by ELISA and normalized to values measured in untreated cells. Data are mean + SE of triplicate determination.

### Statistical analysis

All data are expressed as mean ± SEM, and statistically analyzed using unpaired Student’s t-test. Differences were considered statistically significant when *p* < 0.05.

## Abbreviations

ADR: Adnamycin
CARP-1: Cell cycle and apoptosis regulator 1
CSCs: Cancer stem cells
CFM-4.16: CARP-1 functional mimetics
TNBC: Triple-negative breast cancer
FZD-8: Frizzed receptors 8
LRP-6: Low-density lipoprotein receptor-related protein 6 receptor
CisR: Cisplatin resistant cells
PDX: Patient derived xenografts
ALDH: Aldehyde dehydrogenase
non-CSC: Non-Cancer stem cells (bulk of tumor cells)
